# Potential impact of individual exposure histories to endemic human coronaviruses on age-dependent severity of COVID-19

**DOI:** 10.1186/s12916-020-01887-1

**Published:** 2021-01-12

**Authors:** Francesco Pinotti, Paul S. Wikramaratna, Uri Obolski, Robert S. Paton, Daniel S. C. Damineli, Luiz C. J. Alcantara, Marta Giovanetti, Sunetra Gupta, José Lourenço

**Affiliations:** 1 Department of Zoology, University of Oxford, Oxford, UK; 2 Independent Consultant, London, England; 3 School of Public Health, Tel Aviv University, Tel Aviv, Israel; 4 Porter School of the Environment and Earth Sciences, Tel Aviv University, Tel Aviv, Israel; 5 Department of Pediatrics, Faculdade de Medicina da Universidade de São Paulo, São Paulo, Brazil; 6 Laboratório de Genética Celular e Molecular, Universidade Federal de Minas Gerais, Belo Horizonte, Brazil; 7 Laboratório de Flavivírus, Instituto Oswaldo Cruz Fiocruz, Rio de Janeiro, Brazil

**Keywords:** COVID-19, SARS-CoV-2, Endemic coronaviruses, Cross-reactivity, Immunopathology, Mathematical model, Infectious disease dynamics, Individual-based model

## Abstract

**Background:**

Cross-reactivity to SARS-CoV-2 from exposure to endemic human coronaviruses (eHCoV) is gaining increasing attention as a possible driver of both protection against infection and COVID-19 severity. Here we explore the potential role of cross-reactivity induced by eHCoVs on age-specific COVID-19 severity in a mathematical model of eHCoV and SARS-CoV-2 transmission.

**Methods:**

We use an individual-based model, calibrated to prior knowledge of eHCoV dynamics, to fully track individual histories of exposure to eHCoVs. We also model the emergent dynamics of SARS-CoV-2 and the risk of hospitalisation upon infection.

**Results:**

We hypothesise that primary exposure with any eHCoV confers temporary cross-protection against severe SARS-CoV-2 infection, while life-long re-exposure to the same eHCoV diminishes cross-protection, and increases the potential for disease severity. We show numerically that our proposed mechanism can explain age patterns of COVID-19 hospitalisation in EU/EEA countries and the UK. We further show that some of the observed variation in health care capacity and testing efforts is compatible with country-specific differences in hospitalisation rates under this model.

**Conclusions:**

This study provides a “proof of possibility” for certain biological and epidemiological mechanisms that could potentially drive COVID-19-related variation across age groups. Our findings call for further research on the role of cross-reactivity to eHCoVs and highlight data interpretation challenges arising from health care capacity and SARS-CoV-2 testing.

**Supplementary Information:**

The online version contains supplementary material available at 10.1186/s12916-020-01887-1.

## Background

COVID-19 and its causative agent, SARS-CoV-2, have recently emerged as a global threat to human health, forcing many countries to undertake unprecedented measures to contain its spread. This disease displays a spectrum of illness severity and fatality characterised by a marked age gradient. Typically, infections under 20 years of age display mostly mild or no symptoms, while older individuals are at increased risk of developing severe symptoms, including respiratory failure, multiorgan dysfunction and death [1, 2].

Understanding the determinants of severe symptoms is key to preparedness against COVID-19. So far, cohort studies have identified a number of risk factors for severe illness in comorbidities such as cardiovascular disease, diabetes mellitus and obesity [3–5]. It has also been proposed that viral inoculum size could modulate pathogenicity of SARS-CoV-2 infection [6]. Meanwhile, there have been extensive efforts to calculate age-specific odds of developing clinical and severe symptoms, as well as hospitalisation and fatality rates [7–9]. These outputs have important implications for public health, influencing real-time management and strategic allocation of clinical resources. Nonetheless, apart from a few notable exceptions [10–12], most modelling work assumes that SARS-CoV-2 spreads across an entirely susceptible population both to infection and disease. Consequently, the impact of cross-reactivity between SARS-CoV-2 and other endemic human coronaviruses (eHCoVs), remains largely unexplored in theoretical modelling.

SARS-CoV-2 is the seventh coronavirus known to infect humans. SARS-CoV and MERS-CoV recently emerged from zoonotic reservoirs, while HCoV-229E, HCoV-NL63, HCoV-OC43 and HCoV-HKU1 are endemic to the human population. Infection with eHCoVs is frequent but, contrary to emergent HCoVs, it is usually associated with mild respiratory illness [13]. Typically, the first exposure to any eHCoV occurs early during childhood, but frequent reinfection can occur [14] due to the waning of homotypic immunity [15–18].

So far, a fully mechanistic explanation of COVID-19 severity, that accounts also for the heterogeneous immune landscape in which SARS-CoV-2 spreads, is lacking. T cell and IgG antibody reactivity to SARS-CoV-2 have been observed in non-exposed individuals [19–27], indicating that there is cross-reactivity between eHCoVs and SARS-CoV-2. The role of pre-existing cellular and antibody responses to SARS-CoV-2 remains, however, unclear [28–35]. In this study, we present a parsimonious model of eHCoV co-circulation to explore the effect of these contrasting possibilities on the age distribution of COVID-19 severity. The key assumption is that distinct life-histories of exposure to eHCoVs result in responses of varying effectiveness upon challenge by SARS-CoV-2 and, consequently, distinct clinical outcomes. We assume that the first infection with any eHCoV naturally occurring early in age induces cross-protection against severe COVID-19, but this is reduced in subsequent and frequent eHCoV infections which boost strain-specific responses at the expense of cross-reactive responses. We contrast the results of this model with one where risk of disease is exposure-independent (i.e. determined solely by factors such as immune senescence) and outline the conditions under which both models provide a good fit to the age-specific hospitalisation rates in EU and European Economic Area (EEA) countries and the UK (see the “Availability of data and materials” section for the list of countries examined in this study).

## Methods

### Modelling eHCoVs and SARS-CoV-2 spread

We consider a homogeneously mixed host population of constant size N. The population is endowed with a realistic age profile modelled using a Weibull distribution with scale *θ*_*a*_ and shape *k*_*a*_ [36].

We consider a multi-strain epidemic model with *n* strains. Each strain *i* is characterised by a per-contact transmission probability *β*_*i*_ (*i* = 1, 2, …, *n*) and a daily recovery probability *σ*_*i*_. As reinfection is commonly observed in eHCoVs [18, 37, 38], we assume that the recovered status from strain *i* provides only partial protection against reinfection with the same strain; in particular, we assume that exposure to a previously encountered strain results in infection with probability *ρ*. The case *ρ* = 0 corresponds to complete, life-long immunity upon recovery. For simplicity, we assume that strains do not interact with each other and therefore spread independently. Infection and recovery processes are stochastic and occur in discrete time, with the time unit set to 1 day.

Because eHCoVs display marked annual incidence patterns [39, 40], we add an external sinusoidal forcing *f*(*t*) to transmissibility with period 1 year and intensity *ϵ*:

$$ f(t)=1+\epsilon \cdot \sin \left(2\pi \left(t-182\right)/365\right). $$

If we assume that each individual establishes on average *k* daily contacts, the overall force of infection (FOI) associated to strain *i* is given by:

$$ {\lambda}_i(t)=k\cdot {\beta}_i\cdot f(t)\cdot {I}_i/N,, $$

where *I*_*i*_ is the number of individuals infected with strain *i*.

In this work, we consider *n* = 5 strains; strains labelled *i* = 1, 2, 3, 4 represent eHCoVs, while strain *i* = 5 represents the pandemic human coronavirus (pHCoV), i.e. SARS-CoV-2. eHCoVs are introduced into the system at *t* = 0, while the pHCoV is introduced at a later time *T*_inv_ by infecting 10 individuals chosen at random. We choose *T*_inv_ to be large enough (here we set *T*_inv_ = 160 years) so that by the time the pHCoV is introduced, both population demography and eHCoVs have already reached stationarity. We avoid permanent extinction of eHCoVs by allowing external introductions, which occur at an individual rate *ν*.

### Modelling hospitalisation with heterogeneous risk of severe disease

Let *X* = {*x*_1_, *x*_2_, …, *x*_*M*_} be a list of hosts infected by the pHCoV over a particular time window in a single simulation. In order to select hospitalised cases from *X*, we first draw the total number *m* of these, which is binomially distributed with parameters *M* and *π*. In a second step, we create a list $\overset{\sim }{X}=\left\{{\overset{\sim }{x}}_1,{\overset{\sim }{x}}_2,\dots, \tilde{x}_{m}\right\}$ of hospitalised cases by randomly selecting *m* cases without replacement from *X*, with odds proportional to their corresponding scores *w*_1_, *w*_2_, …, *w*_*M*_.

## Results

### eHCoVs calibration and dynamics

Epidemiological parameters characterising eHCoVs and the pHCoV (SARS-CoV-2) were informed, where possible, from the existing literature. As reinfection is commonly observed in eHCoVs [18, 37, 38], we assume that the recovery provides only partial protection against reinfection with the same strain; in particular, we assume that exposure to a previously encountered strain results in infection with probability *ρ*. The case *ρ* = 0 corresponds to complete, life-long immunity upon recovery. We investigated the impact of the remaining parameters numerically, leveraging available epidemiological knowledge about eHCoVs to guide our analysis. Values defining the life-expectancy distribution were set to simulate age profiles matching average European patterns (simulated age profiles are shown in Fig. S1 of Additional file 1 [10, 41–49]). Model parameters and their values are briefly summarised in Table S1 of Additional file 1, while details about model implementation and calibration are found in Additional file 1.

**Fig. 1:**
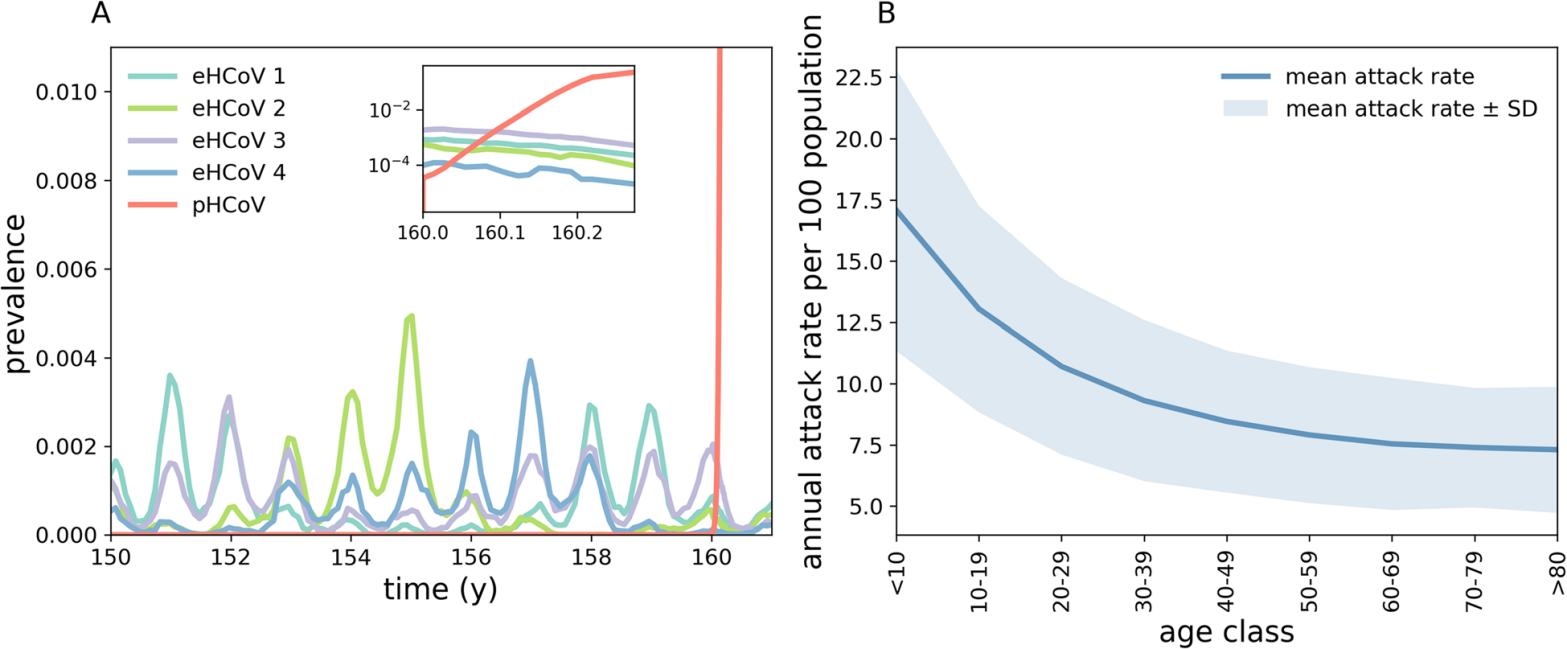
Simulated transmission of eHCoVs. **a** Stochastic realisation of the multi-strain model obtained using baseline parameters. At time *t* = *T*_inv_ = 160 years we randomly infect 10 individuals with the pHCoV (red). Inset: zoom on the first 100 days after the introduction of the pHCoV. **b** Annual attack rate per 100 population by age class for all four eHCoVs. The attack rate decreases and plateaus with age since individuals accumulate immunity through consecutive infections with different eHCoVs

Figure 1a shows typical realisations of our multi-strain model under baseline conditions (parameter values indicated in bold in Table S1 of Additional file 1). The model captures annual patterns of eHCoV spread [49], with seasonal differences in eHCoV epidemics dictated mostly by stochasticity, population turnover and seasonal variation in transmissibility. In addition, Fig. 1b shows that our model captures eHCoVs age-incidence profiles [50]. At time *T*_inv_ = 160 *y* we introduce the pHCoV (SARS-CoV-2) into the host population. As shown in the inset in Fig. 1a, the invading strain rapidly spreads through the population thanks to its antigenic novelty, infecting a large proportion of hosts.

**Fig. 2:**
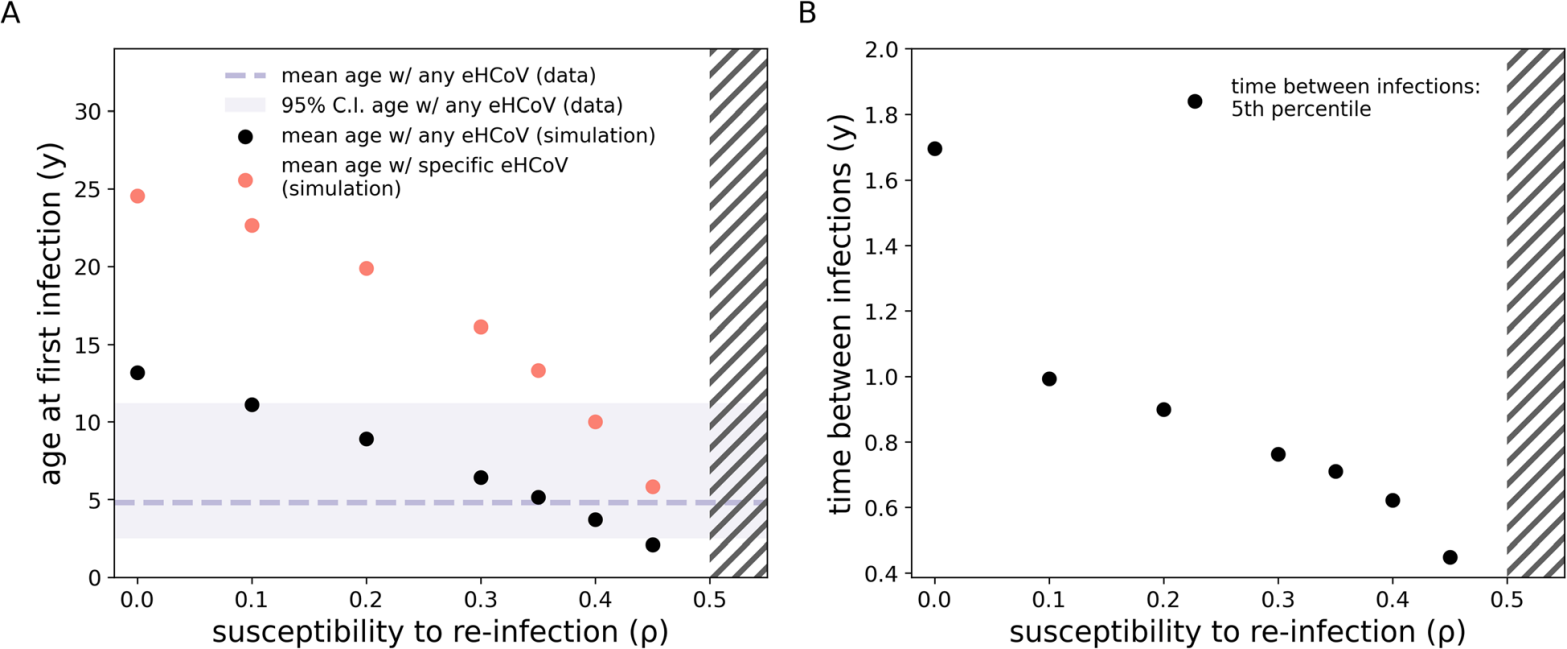
Impact of *ρ* on eHCoV dynamics and pHCoV hospitalisation rates. **a** Mean age at first infection with any eHCoV (black dots) and with a specific strain (red dots) as a function of *ρ*. The dashed line and shaded area represent respectively the mean and 95% C.I. for the age at first infection with any eHCoV obtained from a pooled estimate [50]. **b** 5th percentile of the time (measured in years) between consecutive infections by the same strain. Hatches indicate the *ρ* > 1/*R*_0_ region (for *R*_0_ = 2) where the dynamics are Susceptible-Infected-Susceptible-like (whereas for *ρ* < 1/*R*_0_ we observe epidemic behaviour). It should be noted that for *ρ* = 0 reinfection still occurs in our model because of external introductions, which we have assumed for simplicity to ignore pre-existing immunity to reinfection. Nonetheless, reinfection events induced by external introductions represent only a small fraction of all infection events. Epidemiological parameters are set to baseline values

We set susceptibility to reinfection to *ρ* = 0.35 in order for our model to yield a realistic FOI for eHCoVs. That is, we calibrated *ρ* as to match empirical estimates of the age at first eHCoV infection (Fig. 2) [50], which is given by the inverse of the FOI [51]. In our model, the pHCoV shares the same value of *ρ* as that of endemic strains. This assumption is compatible with recent work finding similar kinetics of antibody responses after both SARS-CoV-2 and eHCoVs infections [52].

We investigated the role of susceptibility to reinfection (*ρ*) on the dynamics of eHCoVs. Increasing values of *ρ* (at fixed *R*_0_) yield a larger force of infection, which in turn affects almost every aspect of eHCoVs’ epidemiology. Figure 2a shows, the effect of *ρ* on the age of first infection with endemic strains: because of the relationship with FOI, larger values of *ρ* yield a younger mean age at first infection. Figure 2a also shows that values of *ρ* in the range [0.1–0.45] yield realistic values for the mean age at first infection with endemic strains, which is estimated to be 4.8 [2.5–11.2 95%C.I.] years globally [50]. The realistic range of values for the mean age of infection with any eHCoV is indicated by a shaded area in Fig. 2a. Consequently, in accordance with previous studies, our analysis rules out the possibility of complete, life-long complete immunity against reinfection by eHCoVs (i.e. *ρ* = 0) [50]. Furthermore, very small values of *ρ* provide unrealistic age-specific incidence profiles. In the extreme case *ρ* = 0 (no reinfection), infections would occur only in older children and young adults, contradicting empirical evidence of eHCoVs infecting older age classes [50]. Finally, we note that values of *ρ* > 1/*R*_0_ cause a shift from an epidemic Susceptible-Infected-Recovered-like behaviour to a stable Susceptible-Infected-Susceptible-like behaviour [53], suggesting values of *ρ* beyond *ρ*_*c*_ (indicated with hatches in Fig. 2) are not epidemiologically plausible in the context of eHCoV dynamics.

Figure 2b also shows that our model reproduces reasonably short times between two consecutive infection events. Specifically, for *ρ* > 0.2 at least 5% of all reinfection events occur within 1 year since the last infection event. Our results agree with previous challenge experiments and cohort studies, which reported short-lived homotypic immunity (< 1 year) against eHCoV reinfection in a minority of individuals [15, 18, 38].

### Modelling age-specific COVID-19 hospitalisation rates under eHCoV exposure dependence

We model individual-level heterogeneities in the probability of developing COVID-19 severe symptoms by assigning to each case a *severity score w*.

Exposure dependence is introduced by:

1 $$ w=\left[a+I\left({n}_{\mathrm{inf}}>0\right)\cdotp \exp \left(\ b\cdotp \left({n}_{\mathrm{inf}}-1\right)\ \right)\right]\cdotp \left[1-\exp \left(-r\cdotp \varDelta {t}_{\mathrm{last}}\right)\right], $$

where *n*_inf_ is the number of previous infections to eHCoVs, *Δt*_last_ is the time since the most recent first infection by any eHCoV and *I*(…) is an indicator function that equals 1 if the condition in the brackets is true and 0 otherwise. The parameter *a* (baseline risk) represents the score in HCoV-naive individuals, *b* represents the variation in severity score after infection with any eHCoV and *r* (waning of cross-protection) is the rate at which eHCoV-priming-induced protection against severe COVID-19 wanes over time. The two terms in square brackets reflect two distinct biological assumptions about the risk of developing COVID-19 severe symptoms following infection by the pHCoV:

1. We assume that primary infections with any eHCoV (immune priming) confer temporary protection against severe infection with SARS-CoV-2: after an individual encounters an eHCoV for the first time, the severity score is reset to 0, but increases progressively with time at rate *r* (waning of cross-protection) back up to the value *a* + exp( *b* · (*n*_inf_ − 1) ). Subsequent infections with the same eHCoV do not provide any additional cross-protection on the assumption that reinfection enhances homotypic responses at the expense of cross-reactivity to SARS-CoV-2. We further remark that subsequent eHCoV infections do not further alter susceptibility to re-infection in this model.
2. The severity score increases exponentially with the number *n*_inf_ of previous infections to any eHCoV to reflect the potential build-up of homotypic immunity superseding heterotypic immunity. The parameter *b* (boosting factor) quantifies the post-infection increment to the score.

In each simulation, we sample a fraction *π* of infected cases without replacement, with severity scores representing sampling weights, and mark them as hospitalised (see the “Methods” section for additional details). For this theoretical exercise, we consider only individuals infected up to 50 days after the introduction of the pHCoV, i.e. those individuals that become infected during the early phase of the epidemic, as a proxy for the time window before containment measures would have a significant impact on the epidemic. *π* is the overall fraction of cases hospitalised and thus represents the Infection Hospitalisation Ratio (IHR) which effectively aggregates multiple factors affecting reporting, e.g. visibility of symptoms, testing efforts, care-seeking behaviour and health care capacity.

**Fig. 3:**
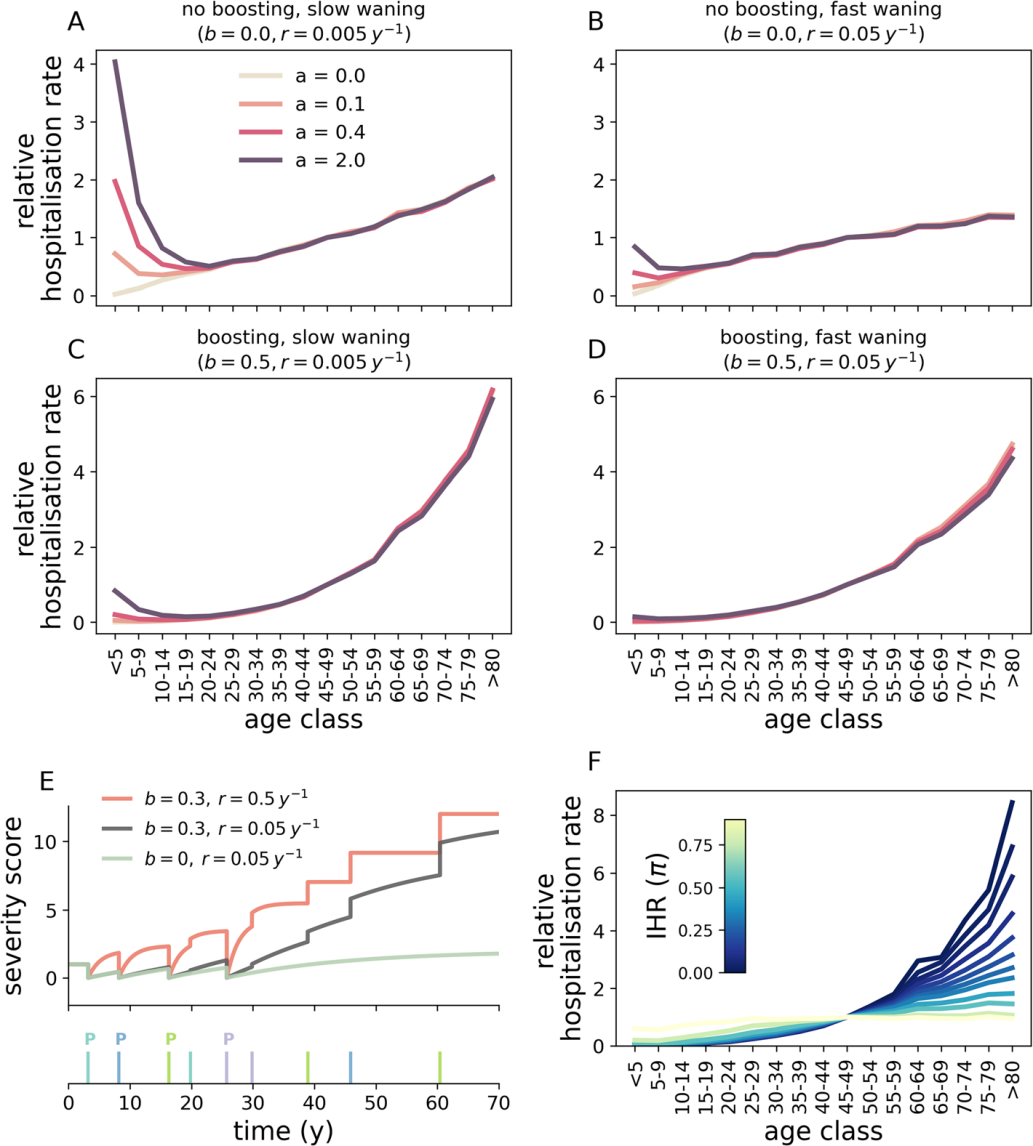
Impact of *b* (boosting factor), *r* (waning of cross-protection), *a* (baseline risk) and *π* (IHR) on age-specific hospitalisation rates. Panels **a**–**d** correspond to different combinations of parameters *b* and *r*. Within each panel, different curves correspond to different values of *a*. Here we assumed that a fraction *π* = 0.1 of all cases are hospitalised. For visualisation purposes, the rate corresponding to the 45–49 years age range is set to one and the remaining rates are re-scaled accordingly. **e** Temporal evolution of the severity score for a single host under different combinations of *b*, *r* and *a* = 1. We considered three scenarios corresponding to no boosting and slow waning of cross-protection (green line, *b* = 0, *r* = 0.05 years), boosting and slow waning of cross-protection (black line, *b* = 0.3, *r* = 0.05 years), boosting and fast waning of cross-protection (red line, *b* = 0.3, *r* = 0.5 years). The lower sub-panel shows the timeline of eHCoV infection events, with each colour corresponding to a different eHCoV. Priming events are denoted with a “P” to distinguish them from secondary infections with the same eHCoV. At birth, the score is identically equal to *a*. The score drops to 0 after encountering a new strain (thicker and taller bars) but increases thereafter at rate *r*. Secondary infections with the same eHCoV (smaller bars) do not provide any additional protection and only increase the score for *b* > 0. In panel **f**, we set *a* = 0.4, *b* = 0.5, *r* = 0.05 years^−1^ and explore *π*. In all panels, epidemiological parameters are set to baseline values. Results are averaged over 50 samplings obtained from each of 5 different stochastic simulations (the impact of stochasticity on hospitalisation rates is further explored in Fig. S2 of Additional file 1)

Age-specific hospitalisation rates are shown in Fig. 3 when varying *a* (baseline risk), *b* (boosting factor), *r* (waning of cross-protective immune responses) and *π* (IHR). Figure 3a–d shows that differences in the combination of these parameters can lead to widely divergent age patterns in hospitalisation rates. First, we note that the hospitalisation risk increases at older ages simply due to the dominance of strain-specific responses over cross-protective responses (i.e. *b* = 0, panels a, b). Second, for *a* > 0, the risk of hospitalisation is not a monotonic function of age. This is because when HCoV-naive individuals become infected with any eHCoV for the first time, their severity score for COVID-19 drops from *a* to 0, as illustrated by time trajectories of individual severity scores in Fig. 3e. Interestingly, several paediatric cohort studies [54, 55] reported a lower frequency of severe COVID-19 in children and teenagers compared to the very youngest (0–5 years), a behaviour that is qualitatively captured by our set of assumptions. It should be also noted that a similar pattern occurs for the risk of fatal outcome [56]. Our model thus provides a “proof of possibility” for biological and epidemiological mechanisms that could result in such disease-related variation across ages. Because the chances of encountering any eHCoV increase rapidly with age, most individuals have already encountered all eHCoVs by the time they reach adulthood. After that point, the average severity score increases at rate *r* (up to *a* + 1 in the absence of boosting), which results in increasing hospitalisation rates. Increasing values of *r* (waning of cross-protection) reduces this effect, since primed individuals revert to their maximal score faster. These observations hold also in the case where *b* > 0 (panels c, d); however, as expected, increasing values of the boosting parameter *b* result in a steeper increase in hospitalisation rates with age.

Figure 3f shows that, given a specific choice of *a*, *b* and *r* (and hence of a function for the severity score), smaller IHR (*π*) values lead to increasingly heterogeneous hospitalisation rates across age ranges. For very small values of the IHR, only those cases with the largest scores are hospitalised. In contrast, larger values of the IHR increase the number of infected cases that are hospitalised, which makes hospitalisation rates increasingly similar to age-specific attack rates. Crucially, this implies that the IHR has a non-linear impact on hospitalisation rates across ages and suggests that differences in IHR, perhaps due to heterogeneous capacity, admission policy and testing efforts, may partially explain inter-country variations in the relationship of age with hospitalisation [57].

**Fig. 4:**
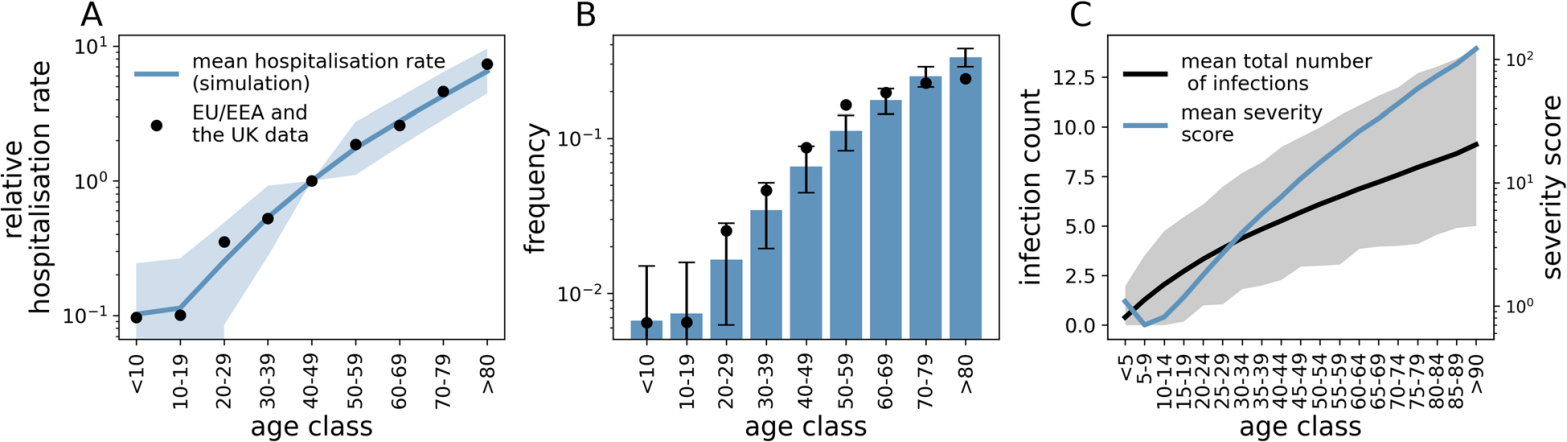
Comparison between model predictions and hospitalisation data for EU/EEA countries and the UK. **a** Simulated hospitalisation rates (blue line) and data (dots). The value corresponding to the 40-49 years age range has been set to 1 for convenience and remaining values have been scaled accordingly. The shaded area indicates the 95% C.I. from simulations. **b** Age distribution of hospitalised cases in EU/EEA countries and the UK (dots) and mean distribution from simulations (bars). Error bars indicate the 95% C.I. from simulations. **c** Mean number of cumulative infections and severity score as a function of age (black and blue lines, respectively), at the time of the introduction of the pHCoV. Shaded area indicates the 95% C.I. from simulations. Here, we set *π* = 0.05, *a* = 1.5, *b* = 0.5, *r* = 0.05 years^−1^. Because our aim is mainly to illustrate the role of disease enhancement and cross-protection, we did not attempt to fit parameters *a* (baseline risk)*, b* (boosting factor) and *r* (waning of cross-protection). Rather, we manually adjusted parameters in order to obtain a good visual agreement between data and simulations. Goodness of fit for chosen parameters was measured at *R*^2^ = 0.98. Results are averaged over 100 samplings from each of 50 different simulations. Other parameters are set to baseline values

In Fig. 4a,b we compare model output and data for EU/EEA countries and the UK obtained from TESSy (ECDC source) [57] under our selected epidemiological parameters (see Table S1) and a value of 5% for IHR, obtained after correcting counts of reported cases for non-uniform attack rates [58], similar to previously reported values [8, 59, 60].

We obtain a good qualitative match to observed trends in hospitalisation rates and the age distribution of hospitalised cases (Fig. 4a, b). In particular, the model seems to capture the relatively low rates observed in individuals aged 0–20 years and the rapid increase in hospitalisation rates after the age of 20. Age variations in severity scores (Fig. 4c) underscore the important role of loss of cross-protection to disease through repeated eHCoVs infections throughout life. Interestingly, Fig. 4c also implies that children in the range 5–19 years are less susceptible to severe symptoms than infants (< 5 years), a pattern previously described for some countries such as Portugal, Italy and the Netherlands (Fig. S3 of Additional file 1) [54]. As explained in the context of Fig. 3, this optimum in protection from severe disease stems from the interplay between losing heterotypic responses in favour of homotypic responses with increasing exposure, and the protective effect of cross-protective immune responses after the first exposure to an eHCoV. Figure S4 and S5 of Additional file 1 further explore sensitivity of our results to susceptibility to reinfection and parameters defining the life-expectancy distribution, respectively.

We also explored the potential for sterilising (ie. infection blocking) heterotypic immunity to account for reduced risk of hospitalisation in children [9]. However, levels of heterotypic immunity required to generate significant levels of protection were not compatible with the observed dynamics of eHCoVs (Fig. S6 of Additional file 1).

### Modelling age-specific COVID-19 hospitalisation rates under age dependence

**Fig. 5:**
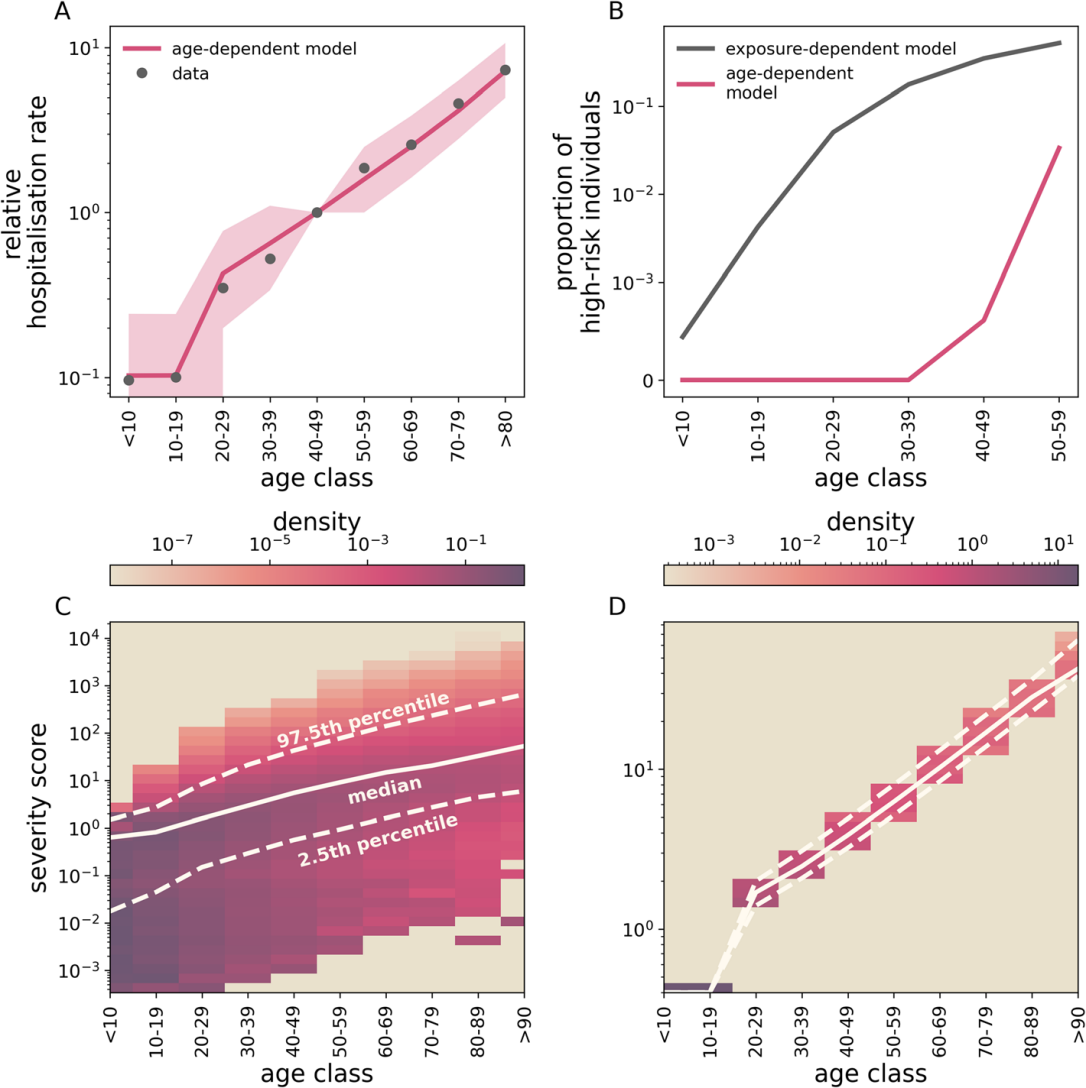
Implications of HCoV-exposure- and age-dependent-severity. **a** A model where disease severity depends only on age (the age-dependent model) is able to explain hospitalisation rates in EU/EEA countries and the UK (dots). Line and filled area represent mean and 95% C.I., respectively. Here, we set *a*_age_ = 0.4, *b*_age_ = 0.052 years^−1^ and *A*_0_ = 20 years, after calibrating the model to hospitalisation data in EU/EEA countries and the UK. Results are averaged over 100 samplings from each of 50 different simulations. **b** Individual risk of developing severe symptoms under exposure-dependent (black) and the age-dependent severity (fuchsia). **c**, **d** Severity score distribution within each age class under exposure-dependant and age-dependent severity, respectively. Solid and dashed lines indicate the median score and the 95% percentile range, respectively. Note that **c**, **d** have different scales. To estimate individual risk in **b**, we first selected $\underset{\_}{N}=3\cdotp {10}^4$ individuals completely at random in a single simulation (that is, we assume uniform infection rates across all age ranges) and then sampled a fraction *π* = 0.05 of these $\underset{\_}{N}$ cases according to the sampling scheme outlined in the “Methods” section. This operation was repeated 2 · 10^3^ times. Finally, we computed the proportion of high-risk individuals as the fraction of sampled cases whose risk is larger than the 25th percentile in the > 60 years age range. In **c**, **d**, the score distribution is computed from a single simulation at the time of the introduction of the pHCoV. We set epidemiological parameters to their baseline values and *a* = 1.5, *b* = 0.5, *r* = 0.05 years^−1^

These data patterns observed for EU/EEA countries and the UK can also be recovered using a model in which disease severity depends only on an individual’s age (the age-dependent model). Briefly, this model assumes that the severity score is constant up to age *A*_0_, but increases exponentially with age thereafter:

2 $$ w(A)={a}_{\mathrm{age}}+{I}_{A>{A}_0}\exp \left(\ {b}_{\mathrm{age}}\cdotp \left(A-{A}_0\right)\right), $$

where *A* is age and *a*_age_, *b*_age_ are positive constants. Estimates of *A*_0_, *a*_age_ and *b*_age_ can be obtained by matching the age-dependent model to hospitalisation data (see Fig. 5a).

Figure 5a shows that such a model is able to capture the observed COVID-19 hospitalisation rates (*R*^2^ = 0.99), under the explicit assumption that individuals younger than *A*_0_ = 20 years are protected from severe symptoms. However, while both HCoV-exposure-dependent and age-dependent mechanisms seem to perform equally well in terms of age aggregated data, they yield different predictions about the individual risk of severe disease (Fig. 5b). In particular, the former model predicts a small but non-negligible proportion of young individuals at high risk of severe COVID-19, whereas the age-dependent model does not. Figure 5c shows that heterogeneity in exposure to eHCoVs results in a fraction of young individuals displaying a severity score comparable to older individuals. In contrast, in the age-dependent scenario younger hosts display a systematically smaller and non-overlapping severity score compared to older ones (Fig. 5d), requiring ad hoc assumptions on why severe disease can sporadically but still significantly happen in this age-group.

## Discussion

Immunopathogenesis of COVID-19 is complex and still far from being completely understood [29]. Here we offer a possible mechanistic explanation of age patterns of COVID-19 severity, based on individual exposure histories to eHCoVs. Our results support the notion that cross-protection induced by exposure to eHCoVs may explain the low frequency of COVID-19 severe symptoms in individuals under 20 years of age [9, 55]. If strengthening of homotypic immunity with repeated exposure interferes with the maintenance of cross-protective responses, this could explain why more immunologically experienced older age classes would be paradoxically more susceptible to COVID-19 disease upon the first infection with SARS-CoV-2. In addition, this argument is also compatible with observed age patterns of SARS-CoV-2 antibody cross-reactivity in non-exposed individuals [23]. We note that in this context, the increase of susceptibility with age due to reduced cross-protection is only a feature of an epidemic caused by a novel strain; if SARS-CoV-2 becomes endemic, there should be sufficient homotypic immunity in older age classes to reduce the severity of a second infection.

Past exposure to eHCoVs may also act to exacerbate symptoms. Antibody-dependent enhancement (ADE) is known to contribute to severity of secondary Dengue infections [61–64] and has been observed also in HIV [65, 66], ebola [67, 68] and influenza [69, 70], having been documented in SARS and MERS [71–75] but its role in the pathogenesis of COVID-19 is still unclear. Anti-spike IgG antibodies have been shown in-vitro to enhance ability to infect immune cells, notably macrophages, and induce the secretion of pro-inflammatory factors for both SARS-CoV [76] and SARS-CoV-2 [77]. Recent studies also indicate widespread T cell reactivity in blood samples obtained during the pre-pandemic period [21, 24, 26]. Pre-trained T cell immunity is likely generated by previous exposure to eHCoVs [78, 79] and is generally thought to promote viral clearance [21, 80]; however, dysregulated CD4 T cell responses have also been shown to contribute to cytokine storm in severe COVID-19 patients [81].

The age distribution of COVID-19 may thus be explained either by the decay of protective cross-reactive responses to eHCoVs or by the accrual of non-protective cross-reactive responses. However, these are unlikely to be the sole drivers of COVID-19 severity. Indeed, we find that an alternative, simple age-dependent model can also match empirical hospitalisation rates across ages (Fig. 5), which could be explained by age differences in angiotensin-converting enzyme 2 (ACE2) expression in the respiratory tract [82]. Interestingly, there have been reports of children presenting no underlying health conditions that developed severe symptoms associated with SARS-CoV-2 infection [55, 83], suggesting additional drivers of COVID-19 severity beyond age. We note that our exposure-dependent model can qualitatively capture this behaviour. The exposure-dependent model allows a small but non-negligible proportion of the young to be at increased risk of severe disease at levels comparable to that of older individuals due to their immunity status (Fig. 5). Further to this point, and although data is limited, the age distribution of severe eHCoVs infections contrasts with the age distribution of severe COVID-19, following a pattern of reduction in severity through repeated exposure (Fig. S7 of Additional file 1). We note that this supports the hypothesis that frequent exposure to eHCoVs throughout age may favour homotypic immune responses to those viruses, thereby compromising the development and persistence of cross-reactive responses that could reduce the severity of disease with the newly emerging coronavirus.

In this work, we considered reported hospitalisation rates as a proxy for severity of symptoms. Alternatively, we could have considered other indicators of disease severity, e.g. rates of severe hospitalisations and fatalities. The former, however, is particularly sensitive to local capacity. The apparent decline in ICU admissions observed in older age-groups, for example, is likely driven by clinical decisions and capacity, rather than by a true decline in disease severity [84]. Fatality rates, on the other hand, are unlikely to provide a robust signal at younger ages because of the small numbers of lethal outcomes in children and teenagers [54]. We focused on data aggregated at the European level, noting that individual countries show a qualitatively similar behaviour (Fig. S3 of Additional file 1). In principle, inter-country variation in age-specific hospitalisation rates might stem from differences in eHCoV circulation patterns. However, in sensitivity exercises, we have shown that heterogeneities in testing and containment efforts, as measured by the IHR (i.e. *π* in our framework), can affect the shape of hospitalisation rates, even under the same biological and epidemiological conditions (Fig. S8 of Additional file 1). Disaggregating these factors from biological mechanisms of pathogenesis will be essential in further research to better understand the human immune responses against SARS-CoV-2 in the context of immunological cross-reactions induced by previous exposure to eHCoVs.

## Conclusion

As we gain a deeper understanding about the landscape of pre-existing cross-reactivity to SARS-CoV-2, it is of paramount importance to understand the clinical and epidemiological impact of past exposure to eHCoVs on the dynamics of COVID-19. Here, we introduce a mechanistic framework that integrates an eco-epidemiological model of eHCoV spread with potential drivers of severe COVID-19. We used this framework to demonstrate a proof of possibility that age patterns of COVID-19-related hospitalisation are self-emergent if strain-specific immune priming and progressive immune specialisation accrue due to recurrent exposure to eHCoVs. Although this work does not commit to a particular immunological mechanism that could sustain the theoretical hypotheses introduced, it can contribute to inform future immunology research on the relationship between eHCoV exposure and the ubiquitous link of age with COVID-19.

## Supplementary Information

**Additional file 1.** Supplementary material composed of supplementary information about the model, Table S1, and Figures S1-S8.

## Data Availability

Data from The European Surveillance System - TESSy, provided by Austria, Croatia, Cyprus, Estonia, Finland, Germany, Hungary, Ireland, Italy, Latvia, Lithuania, Luxembourg, Malta, Netherlands, Norway, Poland, Portugal, Slovakia and UK and released by ECDC. All data is available at cited sources. The views and opinions of the authors expressed herein do not necessarily state or reflect those of ECDC. The accuracy of the authors’ statistical analysis and the findings they report are not the responsibility of ECDC. ECDC is not responsible for conclusions or opinions drawn from the data provided. ECDC is not responsible for the correctness of the data and for data management, data merging and data collation after provision of the data. ECDC shall not be held liable for improper or incorrect use of the data.
