# Supplementary material for: Potential impact of individual exposure histories to endemic human coronaviruses on age-dependent severity of COVID-19
Source: BMC Med. 2021 Jan 12;19:19. doi: 10.1186/s12916-020-01887-1 (PMC7801230; doi:10.1186/s12916-020-01887-1)
Supplement: Supplementary file 1 — Additional file 1. Supplementary material composed of supplementary information about the model, Table S1, and Figures S1-S8. [file 12916_2020_1887_MOESM1_ESM.docx]

**ADDITIONAL FILE 1**

**Potential impact of individual exposure histories to endemic human coronaviruses on age-dependent severity of COVID-19**

Francesco Pinotti^1^, Paul S Wikramaratna, Uri Obolski^2,3^, Robert S Paton^1^, Daniel S C Damineli^4^, Luiz C J Alcantara^5,6^, Marta Giovanetti^5,6^, Sunetra Gupta^1^, José Lourenço^1^

*^1^* *Department of Zoology, University of Oxford, Oxford, United Kingdom; ^2^ School of Public Health, Tel Aviv University, Tel Aviv, Israel; ^3^ Porter School of the Environment and Earth Sciences, Tel Aviv University, Tel Aviv, Israel; ^4^ Department of Pediatrics, Faculdade de Medicina da Universidade de São Paulo, São Paulo, Brazil; ^5^ Laboratório de Genética Celular e Molecular, Universidade Federal de Minas Gerais, Belo Horizonte, Brazil; ^6^ Laboratório de Flavivírus, Instituto Oswaldo Cruz Fiocruz, Rio de Janeiro, Brazil*

**1) Model parameters**

**Table S1** Summary of model parameters and values explored. Baseline values are indicated in bold.

| **Parameter name** | **Description** | **Values explored** |
| --- | --- | --- |
| $\beta_{i} i=1,2,3,4,5$ | Strain-specific per-contact transmissibility | **0.1** (${day}^{-1}$) (for eHCoVs,$i=1,2,3,4$)  $\beta_{5}$adjusted according to average number of contacts |
| $\sigma_{i}$ | Strain-specific recovery rate | **0.1**(${day}^{-1}$) |
| $\rho$ | Susceptibility to reinfection | 0, 0.1, 0.2, 0.3, **0.35**, 0.4, 0.45 |
| $k$ | Average number of daily contacts | **2** |
| $\epsilon$ | Seasonal forcing magnitude | **0.15** |
| $N$ | Population size | **300000** |
| $\nu$ | Introduction rate for eHCoVs | **1.1** ${10}^{-6}$ (${day}^{-1}$) |
| ($\theta_{a}$,$k_{a}$) | scale and shape of lifetime distribution | (80 y, 10), (**90** y**, 12**), (100 y, 50) |

**2) Additional details about model calibration**

Epidemiological parameters characterizing eHCoVs and SARS-CoV-2 were informed, where possible, from the literature. We investigated the impact of the remaining parameters numerically, leveraging available epidemiological knowledge about eHCoVs to guide our analysis.

In this work we consider a neutral epidemiological scenario in which eHCoVs share the same epidemiological parameters, which include $\beta_{i}=\beta=0.1 {day}^{-1}$(transmission potential) and $\sigma_{i}=\sigma=0.1 {day}^{-1}, i=1,2,3,4$ (infection period) [41]. We set the average (contact) degree to $k=2$, so that the basic reproductive number of eHCoVs is $R_{0,i}=\beta k/\sigma=2, i=1,2,3,4$, in line with other epidemiological studies [10]. We assume that SARS-CoV-2's transmissibility is such that $R_{0,5}=2.5$ [42], and set its infection period to 10 days [43, 44].

We set the strength of seasonal forcing to$\epsilon=0.15$, which amounts to a 30% relative difference between the minimum and maximum values for $R_{0}$during a single year. This choice is compatible with previous studies, which estimated a 20-30% reduction in $R_{0}$ between summer and winter [10, 45]. In [45]**,** a mild seasonal forcing was found to be compatible with eHCoV incidence data in Sweden, under the hypothesis that the rate of external introductions was small. Here, we assume that individuals become infected due to external infections/importations at a rate $\nu=1.1 \cdot{10}^{-6} {day}^{-1}$(equivalent to 10 introductions per month in a population with $N=3\cdot{10}^{5}$individuals).

In order to work with data on COVID-19 hospitalisation rates in EU/EEA and UK, we set the values of $\theta_{a}$and $k_{a}$ defining the life-expectancy distribution, so that simulated age profiles match the average European age (simulated age profiles are shown in Fig. S1). This yields $\theta_{a} =90 y$ and $k_{a}=12$.

**3) Additional figures**

**
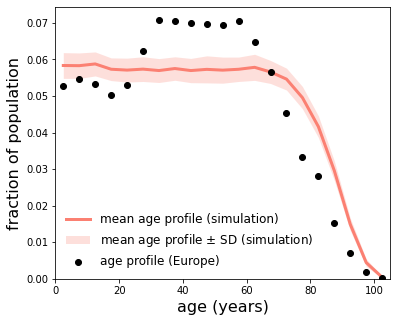
**

**Fig. S1** Age distribution in Europe and in simulations. Solid line and shaded area represent the mean profile and one standard deviation, respectively. The last dot contains individuals aged >100 years. As explained in the main text, we consider a Weibull lifetime distribution with scale $\theta_{a}$ and shape $k_{a}$. Thus, an individual aged $A$ years has a probability $({(A+1)}^{k_{a}}-A^{k_{a}})/\theta_{a}^{k_{a}}$ of dying in a given year. Demographic data for Europe was taken from [46].


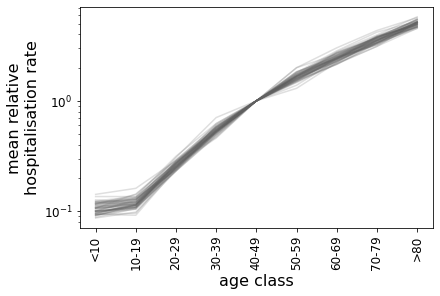


**Fig. S2** Impact of model stochasticity on mean hospitalisation rates due to infection with the pHCoV. Each curve corresponds to a different realisation of the stochastic model (50 simulations in total). For each simulation we averaged hospitalisation rates over 100 samples of hospitalised cases. We set $a = 1.5$*,* $b = 0.5$,$r = 0.05 y^{-1}$*,* and $\pi= 0.1$. Epidemiological parameters are set to baseline values. Rates have been rescaled by the value corresponding to the 40-49y age class.


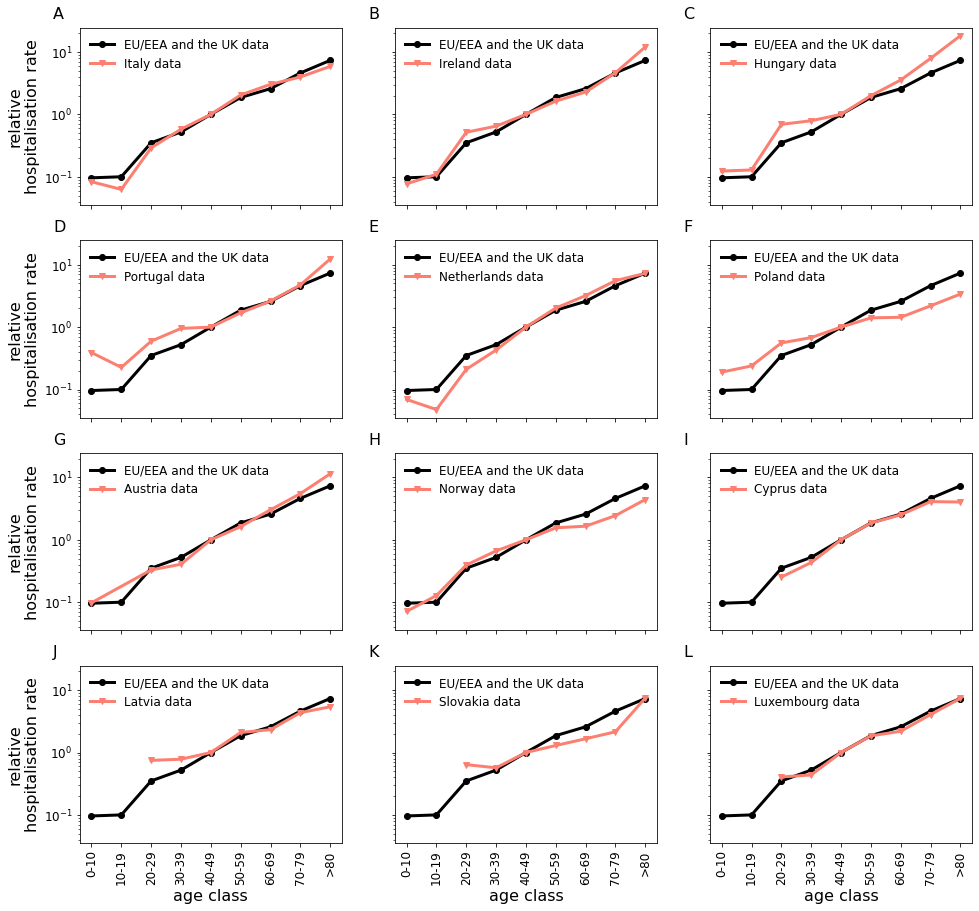


**Fig. S3** Reported hospitalisation rates for some European countries. In particular, we selected the countries with the most complete records from the TESSy database. Within each panel we have also shown the same data, but aggregated at the European level (black line). Rates have been rescaled by the value corresponding to the 40-49y age class. Age distributions of European countries were obtained from [7].


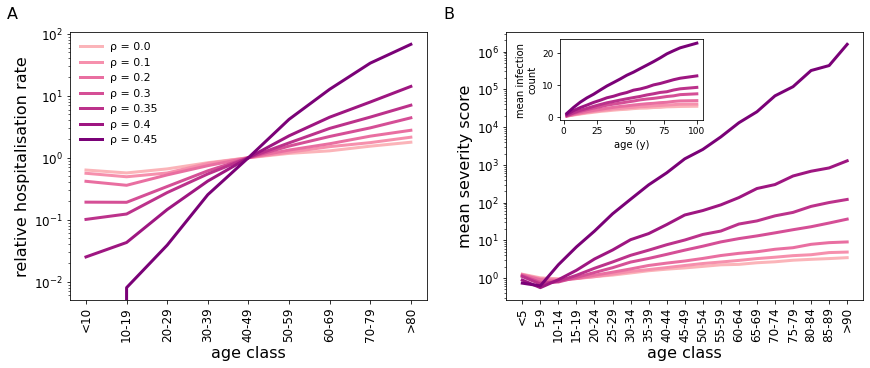


**Fig. S4** Impact of $\rho$ on pHCoV hospitalisation rates. (A) pHCoV age-specific hospitalisation rates for different values of $\rho$. The value corresponding to the 40-49y age range has been set to 1 for convenience and remaining values have been scaled accordingly. (B) Mean severity score as a function of age. The inset in (B) shows the mean cumulative number of infections by eHCoVs as a function of age. Parameters are the same as in Fig. 4 in the main manuscript. Results are averaged over 100 samplings from each of 5 different simulations.


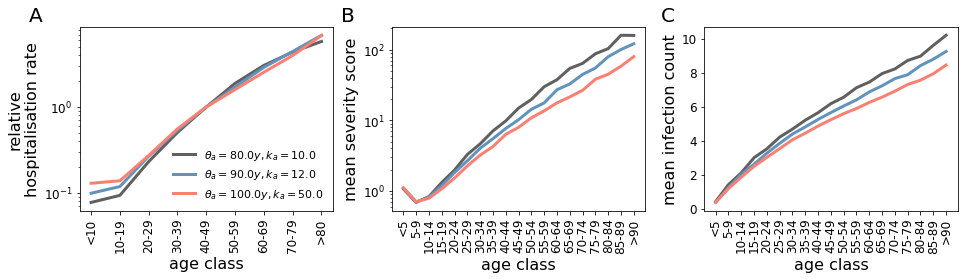


**Fig. S5** Impact of mortality on age-specific hospitalisation rates, severity score, and cumulated infections to eHCoVs. Hospitalisation rates, severity and cumulated infections to eHCoVs are shown in panels (A), (B) and (C), respectively.Here, we considered three different demographic scenarios corresponding to different choices of parameters $\theta_{a}$ and $k_{a}$. Black, blue, and red curves correspond respectively to a mean age that is smaller, equal or greater than the European one ($\theta_{a}=90y$, $k_{a}=12$). Results are averaged over 100 samplings from 5 different simulations. Other parameter values are the same as in Fig 4. (A,B) show that the lower the mean age, the more heterogeneous the hospitalisation rates and severity score profile. Also, the younger population is characterised by the steepest increase in accumulated counts of eHCoV infections (C). This is expected, since the faster turnover of hosts increases the FOI and hence the rate of new exposures to eHCoVs. On such a basis, it is tempting to speculate that further lower mean age could lead to even more heterogeneous patterns of hospitalised cases. However, it is important to note that in the real world, different demographic profiles are also associated with different social and epidemiological contexts. These differences might potentially affect the IHR [47, 48], which we kept fixed across all three populations.


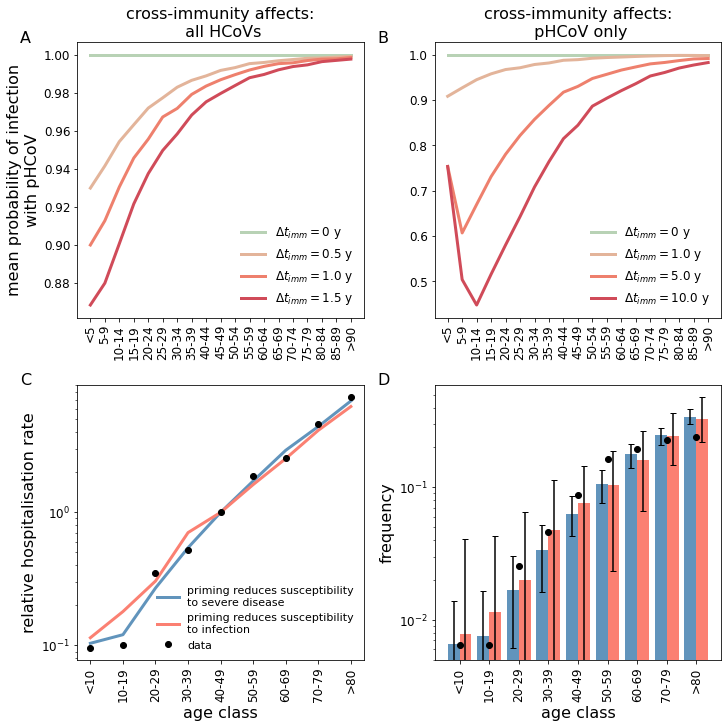


**Fig. S6** Role of reduced susceptibility to infection after priming by eHCoVs. (A,B) Mean age-specific susceptibility to infection with the pHCoV. We assume that after any priming event, susceptibility to heterotypic infection is equal to $\rho$ for a duration $\Delta t_{imm}$. Panel (A) shows that for $\Delta t_{imm} \leq1.5 y$ pan-HCoV cross-protection leads to a minor reduction in susceptibility towards the pHCoV. On the other hand, larger values of $\Delta t_{imm}$would lead to unrealistic eHCoV dynamics. We then considered the less biologically relevant scenario in which eHCoVs induce a cross-immune response towards the pHCoV only (that is, cross-immunity does not affect eHCoV dynamics) (B). In panels (C,D) we show the effect of this last mechanism (with $\Delta t_{imm}=10 y$) on hospitalisation rates and the age distribution of hospitalised cases (red), respectively. Here, we used $a=1.5$ and $b=0.5$ and set $r=\infty$ so that reduced susceptibility to infection is the only protective mechanism induced by priming events. Hospitalisation rates in EU/EEA countries and the UK are shown as well (dots). For the sake of comparison, panels (C,D) also show analogous results for the case of protection against symptoms, similarly to Fig. 4 in the main manuscript (blue), with parameters $a=1.5$, $b=0.5$ and $r=0.05 y^{-1}$. In (C) hospitalisation rates were rescaled by the value corresponding to the 40-49y age range. In (D), the larger error bars observed for the model with protection from infection are the consequence of the smaller numbers of hospitalised cases. In all panels, model parameters are set to baseline values and results have been averaged over 100 samplings from each of 5 different stochastic simulations, obtained using $\pi=0.05$, $a=1.5$, $b=0.5$.


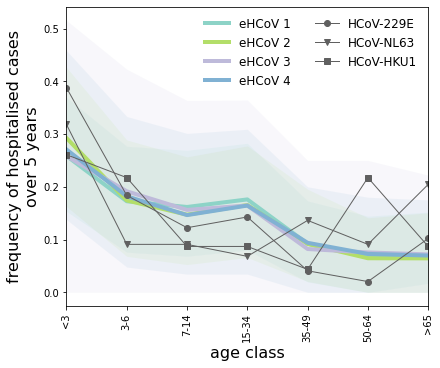


**Fig. S7** Age distribution of severe infections due to eHCoVs. Black lines represent data relative to HCoV-229E (circles), HCoV-NL63 (triangles) and HCoV-HKU1 (squares), collected in a 5-year-long longitudinal study in Guangzhou, China [10]. Data about HCoV-OC43 could not be extracted but showed a qualitatively similar pattern. Coloured lines and shaded areas represent strain-specific mean age distribution and its 95% C.I., respectively, computed from 300 samples from a single realisation of the individual-based model. For this experiment, we collected details (i.e. number and timing of past infection events) about individuals infected by any seasonal HCoV during a 5-year long time period starting at $t=150 y$. From this host pool, we selected a proportion $\pi=0.001$ of hospitalised cases according to the same sampling procedure outlined in the Methods. We assigned severity scores according to Equation (1) in the main manuscript, under the additional assumption that secondary infections with the same strain do not cause severe disease (i.e. $w=0$). We set $a=15$, $b=0.5$ and $r=0.01 y^{-1}$. Other parameters are set to baseline values.


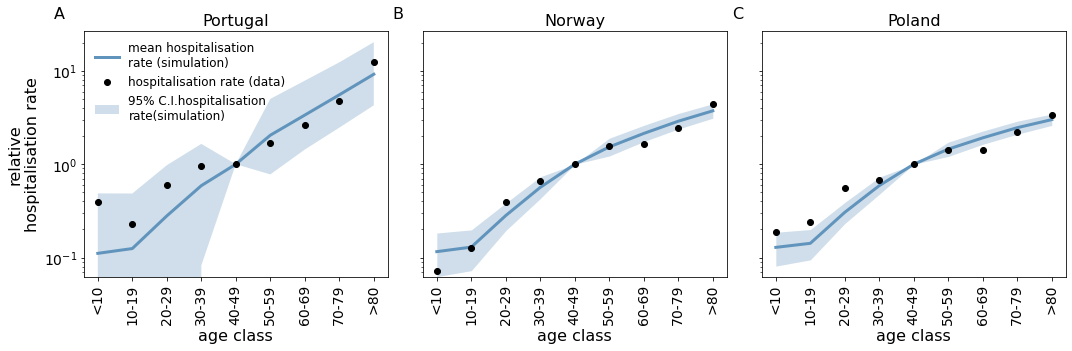


**Fig. S8** Comparison of model output with country-specific data. Panels show hospitalisation rates for Portugal (A), Norway (B) and Poland (C) (dots), along with mean hospitalisation rates from simulations (line) and their error (shaded area indicates 95% C.I.). The value corresponding to the 40-49y age range has been set to 1 for convenience and remaining values have been scaled accordingly. Here, we show that variations in $\pi$ can partially explain differences in hospitalisation rates between countries. To address this point, we used the same simulation data as in Fig 4 and the same values of parameters $a$, $b$ and $r$, while varying $\pi$. In particular, we used $\pi=0.015, 0.18, 0.25$ for Portugal, Norway and Poland, respectively. We note that model's uncertainty becomes smaller as $\pi$ increases. We averaged results over 100 samplings from each of 50 different simulations. Age distributions of European countries were obtained from [46].
